# Variable stressor exposure shapes fitness within and across generations

**DOI:** 10.1038/s41598-025-87334-8

**Published:** 2025-01-29

**Authors:** Marcus Lee

**Affiliations:** 1 Aquatic Ecology, Department of Biology, Lund University, Lund, Sweden; 2 Department of Biology, University of Texas at Arlington, Arlington, USA

**Keywords:** Environmental heterogeneity, Life-history trade-offs, Stressor delivery, Multigenerational, Clonal variation, *Daphnia*, Evolutionary ecology, Freshwater ecology

## Abstract

Environmental variation has long been considered a key driver of evolutionary change, predicted to shape different strategies, such as genetic specialization, plasticity, or bet-hedging to maintain fitness. However, little evidence is available with regards to how the periodicity of stressors may impact fitness across generations. To address this gap, I conducted a reciprocal split-brood experiment using the freshwater crustacean, *Daphnia magna*, and an ecologically relevant environmental stressor, ultraviolet radiation (UVR). I exposed one group to constant and another group to fluctuating UVR conditions. Despite receiving the same dose of UVR, the first experimental generation displayed significant treatment-by-genotype interactions with respect to survival and reproductive output, as well as a delayed reproductive maturity under fluctuating UVR conditions. In the following experimental generation individuals exposed to fluctuating UVR exhibited higher fitness than those in a constant UVR regime. The ancestral conditions, i.e., maternal environment, however affected the survival probability and reproductive output, but did not significantly influence the maturation date. Overall, I demonstrate that the delivery of a stressor, not just its intensity, can have profound fitness consequences across generations, with important implications for seasonal succession of genotype–phenotype patterns in natural environments.

## Introduction

An organism’s phenotype reflects the developmental interplay between past and current environments^1^. Selection embeds information of past environments within the genotype^2^, and phenotypic plasticity allows for the assimilation of current environmental cues into locally adapted phenotypes^3^. Moreover, non-genetic inheritance mechanisms, such as maternal effects, have repeatedly been shown to contribute to the individual’s phenotype^4,5^. Despite the clear beneficial potential of integrating information across recent ancestry, from environmentally induced sources^6,7^, the adaptive nature of transgenerational plasticity (TGP) is not always evident^8–11^. In part this can be due to environmental unpredictability violating one of the fundamental assumptions of plasticity which is that organisms have the ability to accurately detect and forecast the effects of changing environmental conditions^12^.

This unpredictability may arise from the failure to detect stressors, unreliable cues, or stressors that vary at scales that preclude the advantages conferred through plasticity^13^. In response to perceived unpredictability, an alternative strategy that may evolve is bet-hedging. This is essentially the reduction in mean fitness at the individual level that then decreases the variation in fitness across generations^14^. In other words, so long as some individuals are able to reproduce in a given generation, then unpredictable fluctuations are unlikely to eliminate a genotype from the population. Although bet-hedging has been shown to be able to evolve through many models^14,15^, the ability to produce unpredictable experimental regimes have limited the number of empirical examples^16–18^.

Given that environmental variations then clearly have the potential to alter the evolutionary strategy utilised^19^, many studies have examined the effects of stressor introduction during specific developmental stages on fitness within and between generations^9,20,21^. However, these experiments have typically focused on the timing of stressor introduction rather than the temporal variability of stressors, particularly of those with a more ephemeral nature. Thus, our understanding of the responses of organisms to highly fluctuating environments remains limited^22^. Research has explored the impacts of temperature variability^23,24^, with some papers suggesting prior information of unpredictability ‘primed’ the organisms’ physiology to be better able to cope with extremes in temperature^25^. However, due to the complexity of the underlying physiological mechanisms that modulate the expressed phenotype, clear directional hypotheses of the effects of variability are often more challenging, as made clear by a recent meta-analysis which found longevity to decrease under fluctuating temperatures^26^. Even less knowledge is available about the effects of variability in other biotic and abiotic stressors. This is of increasing importance in light of the predicted increase in environmental variability due to climate change^27^, which directly effects a variety of abiotic factors such as temperature, precipitation and solar ultraviolet radiation (UVR).

In aquatic systems, UVR is a common and highly variable stressor for many organisms. It varies over seasonal scales, diel scales and even at shorter time scales, such as rapid changes in cloud cover (Supplementary Fig. S1). Zooplankton may be the best studied group of aquatic organisms in relation to the effects of UVR, with common negative consequences including reductions in longevity^28^, lower reproductive output^29^, reduced growth rates^30^ and even increased respiration rates^31^. In response to the direct negative consequences of UVR, zooplankton have a suite of phenotypic responses including the upregulation of photoprotective compounds, such as melanin or carotenoids^32,33^, alternative antioxidant stress responses^34^ or behavioural avoidance strategies such as diel vertical migration^35,36^. The relative expression of responses to a threat, such as UVR, will then inevitably depend on the current ecological trade-offs (such as predation risk and foraging opportunities)^37,38^ coupled with the information about past environments.

One of the most extensively studied zooplankton species is *Daphnia magna*. They represent a key species in shallow lakes and ponds across the northern hemisphere. Furthermore, they have become an invaluable model for all manner of evolutionary and ecological research questions due to their amenability to lab-based studies, in part because of their cyclical parthenogenetic mode of reproduction^39^. In particular, they are a powerful model for investigating transgenerational responses to stressors^4,9,40,41^, including towards UVR^10^. Despite the replete literature on threat response in the genus *Daphnia*, and the long history of them being studied in relation to UVR both within and across generations, very few studies consider the variable nature of the stressor. Those that do are limited to short-term exposure, or within-generation studies^42,43^. Furthermore, *Daphnia* often exhibit diverging responses due to the variation between genotypic lines^43,44^. As a result, we may make predictions regarding population viability that over- or underestimate a populations´ capacity to adapt. Therefore, it is crucial to understand empirically how organisms respond to either consistency or fluctuations in this environmental variable both within and across generations in multiple genetic lines.

**Fig. 1:**
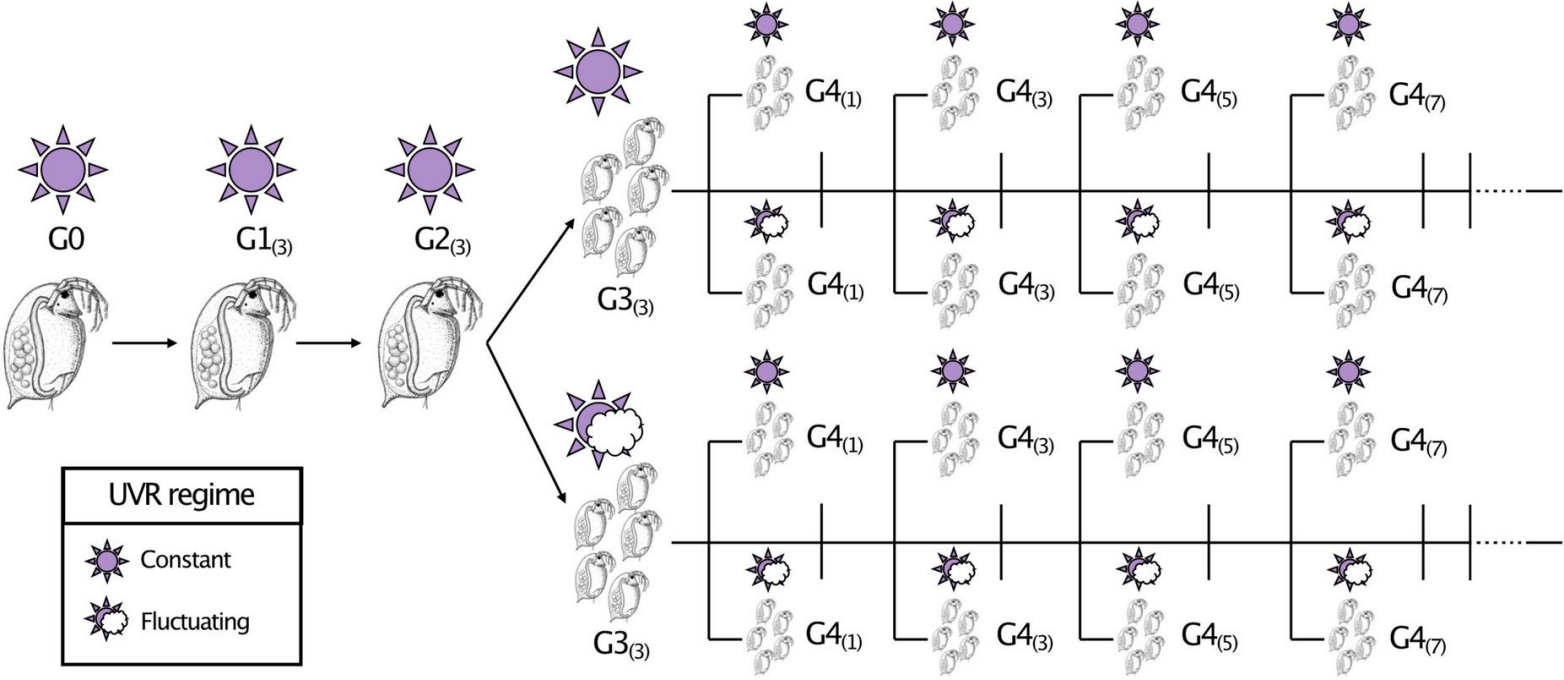
Schematic of the experimental split brood design imposed on each of the three genotypes. Generation is denoted by the letter G and the number in the brackets denotes the maternal clutch from which the focal generation were obtained. Each individual from generation G3 and G4 was followed for the entirety of their lifetimes, which is denoted by the horizontal lines. Each vertical intersecting line represents a clutch, of which only four were followed from G3. All other clutches were removed and measured.

In order to address this gap in the available knowledge, I conducted a reciprocal split-brood experiment (Fig. 1) to investigate the responses of *D. magna* to constant and fluctuating UVR regimes for multiple generations. Theory predicts within-generation predictable variation should select for plastic responses to maintain fitness under stress. Building upon previous research, which demonstrated behavioural shifts in activity due to UVR fluctuations have negative reproductive consequences within a generation^43^, I hypothesised that constant exposure would yield fitness advantages within a generation. Specifically, I predicted earlier reproductive maturity and higher lifetime reproductive output, despite the trade-off with a shortened lifespan. Furthermore, given environmental consistency across generations i.e., maternal matching, is predicted to confer advantages^41,45^, I hypothesised that offspring experiencing the same environment as their mother would gain a fitness advantage in comparison to those that are mismatched. In this generation, I predicted the fitness advantage would manifest as greater survival in matched maternal environments.

## Methods

To ensure genotypic variation, *Daphnia magna* were collected from three source populations located in Southern Sweden. These localities were Lake Fnifahosjön (N 55.73907, E 13.20968; genotype ‘N’), Lake Bysjön (N 55.675393, E 13.545363; genotype ‘D’) and a Sydvatten pond (N 55.661140, E 13.541958; genotype ‘P’). The genotypes were cultured in the laboratory for over 50 generations at 18 °C, a 16:8 light:dark photoperiod, fed with a green alga live culture (*Tetradesmus obliquus*; formerly *Scenedesmus obliquus*^46^) and maintained at a density of ca. 100 individuals per litre. To establish the experiment (G0), one individual female per genotype was isolated and placed into separate 100 ml jars filled with 80 ml of Artificial *Daphnia* Medium (ADaM)^47^ and 240,000 cells ml^−1^ of *T. obliquus*. These jars were then placed under the same conditions with the addition of ultraviolet lamps (Sylvania F36W/GRO; 70 ± 10 μW cm^−2^) at ecologically relevant conditions of UVR (Supplementary Figs. S2–S3). These lamps were turned on for six hours each day between 10:00 and 16:00 and constituted a constant UVR exposure treatment.

This common-garden condition was maintained for two subsequent generations to remove potential maternal effects of moving to an increased UVR environment. It is important to highlight here, as copious research has investigated the stress response of UVR^31,33,38,48–51^, I have no treatment without exposure to UVR. My focus is the variation of the stress, therefore all differences detected will represent changes in the consistency of the stressor, and not dosage differences. From the moment that G0 individuals were isolated, every individual was transferred to new media and fresh food every other day. Furthermore, to ensure food availability and reduce any competition between either the mother and offspring, or between siblings, every individual was isolated and transferred to new media on the day that a brood was released, even if the mother had been transferred the day prior.

The experiment began when the G2 mothers had produced ten clonal offspring from their third brood. This brood was then divided into two treatments ‘Constant’ (as described above) and the ‘Fluctuating’, generating five replicates per treatment per genotype in generation G3. The fluctuating treatment also experienced six hours of UVR, however the schedule oscillated between on and off in 15-min intervals over a 12-h period during the photoperiod. To ensure that the oscillations themselves were unpredictable, I created multiple schedules each with six hours of UVR exposure, then alternated between the schedules randomly every 1–4 days (Supplementary Fig. S4). This scheduling prevented anticipatory modifications between broods and guaranteed that before maturity, each *Daphnia* in the fluctuating treatment was exposed to a variety of UVR schedules. Within both treatments the position of each individual was randomised to ensure that the location within the climate chamber did not influence the results.

To address how the variations of an environmental stressor may affect the life-history of *Daphnia*, I quantified longevity, reproductive maturity, as defined by the first day at which eggs were visible in the brood pouch^52^, and total reproductive output over each individual´s lifetime. Every individual was checked each day at the same time (± 0.5 h). In order to also take into account the offspring’s ‘fitness’, I repeated this design for a second generation using a split brood design. Specifically, I took the first, third, fifth and seventh broods forward to the next generation as to maximise the number of individuals in generation G4 while taking the other broods for size measurements of neonates (data not shown here). When the broods contained six or more offspring, each individual was randomly allocated to either the constant or fluctuating treatment group, generating four ‘Ancestry : Treatment’ combinations. If a brood had fewer than six individuals, then all individuals were removed from the analysis as to prevent unduly different sample sizes (see Table S1 for a breakdown of samples sizes per analysis).

### Statistical analysis

All analyses were performed using R v.4.4.2^53^. The two experimental generations (G3 and G4) were analysed independently. All models were subject to stepwise backward selection, removing any non-significant interaction terms. Post hoc tests were employed to investigate significant models, and *p*-values were adjusted using the Benjamini–Hochberg method for multiple comparisons, thereby decreasing the false discovery rate. Statistical figures were subsequently produced using the package ggplot2 v.3.5.1^54^.

In order to assess how different categorical treatment groups affect lifespan, I utilised a Cox proportional hazard model using the *coxme* package^55^. As all individuals were followed for their lifetime, none were censored. Treatment, genotype and their interaction were used as the independent variables in the model for the G3 generation. The G4 generation was modelled using treatment, ancestral treatment, and their interaction as independent variables. When including genotype into the model, the assumptions of heteroscedasticity are violated. Therefore, I modelled each genotype separately. To account for the possibility that particular mothers invest differentially in offspring, the G4 generation was analysed using a mixed effects model with Mother ID as the random factor.

To examine the effects of treatment, genotype and their interaction on individual reproductive success (the total number of neonates produced) of generation G3 I utilised a two-way ANOVA. Due to the large proportion of zeros within generation G4, this generation was analysed through a zero-inflated generalised linear mixed model with a Poisson error distribution using the ‘*glmmTMB*’ package^56^. Furthermore, due to the underrepresentation of genotypes ‘N’ and ‘P’, genotype was not included in this model. Treatment, ancestral treatment, and their interaction were assigned as fixed factors and the mother ID as a random effect. The assumptions for this model were confirmed using the package ‘*DHARMa*’^57^.

Age of reproductive maturity, as a numerical count, was analysed using a linear model for generation G3 with treatment, genotype and their interaction as independent variables. In order to account for the mother ID as a random factor in generation G4, I analysed age of reproductive maturity with a linear mixed effects model, using the ‘*lme4*’ package^58^. This meant treatment, ancestral treatment, the interaction between current and ancestral treatment were modelled as fixed effects and mother ID as the random effect. Similar to the analyses above, genotype was not included in this model due to underrepresentation of reproductively mature individuals from 2/3 of the initial genotypes. To analyse this disparity in the frequency of reproductive and unreproductive individuals, i.e., the reproductive status, I utilised a Cochran-Mantel–Haenszel test, which is an extension of a Chi-Squared, to explicitly test if this is dependent on genotype and ‘Treatment x Ancestry’ combination.

## Results

*Daphnia magna* exhibit distinct life history responses to variation in UVR stress. Notably, significant interactions were observed in generation G3 for both survival and reproductive output. However, these interactions did not align with predictions based on prior experiments, nor the maternal matching hypothesis. Instead, the results suggest evidence for carryover effects of UVR variation across generations. Below, I provide a detailed account of the results for survival, reproductive output, and reproductive maturity, organized by generation.

### Generation G3

**Fig. 2:**
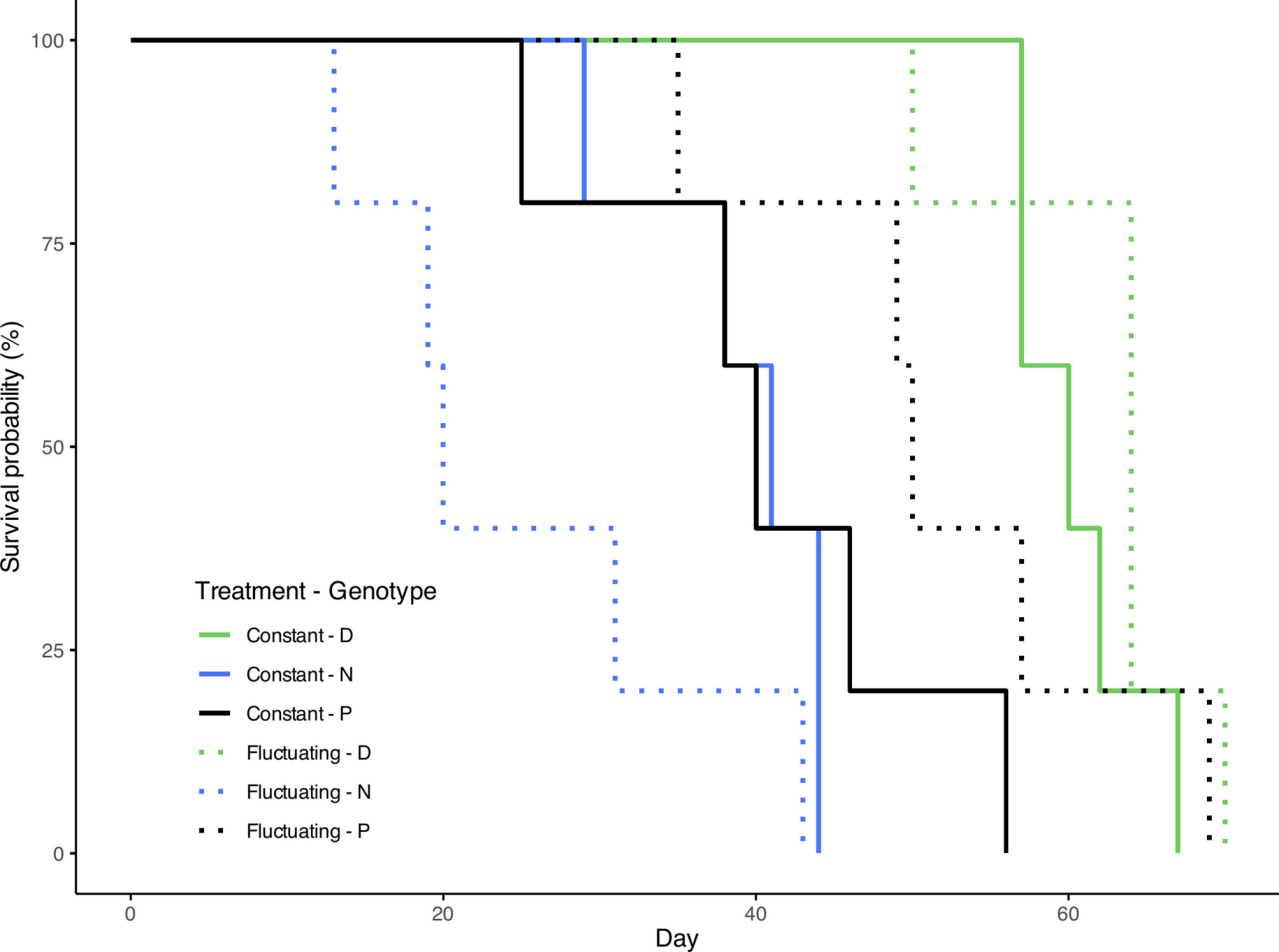
Survival curves of generation G3. Colours denote the genotype and the line type indicates the treatment group.

The interaction between genotype and treatment significantly impacted the survival probability (Table 1). Specifically, genotype P exhibited 3.5 times lower survival in a constant UVR regime, however when in a fluctuating environment they performed similar to genotype D at only 1.13 times higher risk of dying. Under constant stress genotype P were 3 times less likely to survive than genotype D but 12.6 times more likely than genotype N (Fig. 2; Table 2).

**Table 1:** Results of Cox proportional hazard model for survival, and the linear models for maturity and reproduction of generation G3 respectively.

Generation	Dependent variable	Explanatory variable	χ^2^	d.f	*p* value
G3	Survival	Genotype	0.77	1	0.38
		**Treatment**	**13.82**	**2**	**0.001**
		**Treatment × Genotype**	**8.16**	**2**	**0.017**
	Reproduction	**Genotype**	6.51*	**2**	**0.006**
		Treatment	0.17*	**1**	0.69
		**Treatment × Genotype**	4.15*	2	**0.028**
	Maturity	**Genotype**	18.98*	**2**	** < 0.001**
		**Treatment**	6.71*	1	**0.016**
		Treatment × Genotype	2.07*	**2**	0.15
G4	Survival^†^	**Treatment**	**7.64**	**1**	**0.006**
		**Ancestry**	**4.83**	**1**	**0.028**
		Treatment × Ancestry	0.23	1	0.63
		Mother ID	Variance = 0.087; standard deviation = 0.295		
	Reproduction	**Treatment**	**43.37**	**1**	** < 0.001**
		**Ancestry**	**8.58**	1	**0.003**
		Treatment × Ancestry	0.96	1	0.33
		Mother ID	Variance = 0.018; standard deviation = 0.136		
	Maturity	**Treatment**	**6.34**	**1**	**0.012**
		Ancestry	0.01	**1**	0.91
		Treatment × Ancestry	0.17	1	0.67
		Mother ID	Variance = 0.47; standard deviation = 0.68		

Mixed effects versions of the same models were employed to analyse the G4 generation with the Mother ID serving as the random effect. Significant explanatory variables (α < 0.05) are emboldened

*Values shown here relate to the *F* statistic and not to χ^2^

^†^This analysis involves only genotype ‘D’

**Table 2:** Contrasts of survival analysis performed on generation G3 with.

Treatment: Genotype contrast	Hazard ratio	S.E	z statistic	*p* value
Constant : D–N	**4.2**	**0.45**	**3.20**	**0.003**
Constant : D–P	**3.01**	**0.39**	**2.86**	**0.008**
Constant : N–P	**12.64**	**0.76**	**3.36**	**0.003**
Fluctuating : D–N	**5.21**	**0.65**	**2.52**	**0.018**
Fluctuating : D–P	0.87	0.58	-0.23	0.81
Fluctuating : N–P	**8.28**	**0.66**	**3.20**	**0.003**
Constant–Fluctuating : D	0.74	0.34	-0.87	0.43
Constant–Fluctuating : N	**29.48**	**0.85**	**3.97**	** < 0.001**
Constant–Fluctuating : P	**3.55**	**0.60**	**2.09**	**0.047**

Statistically significant differences are emboldened.

**Fig. 3:**
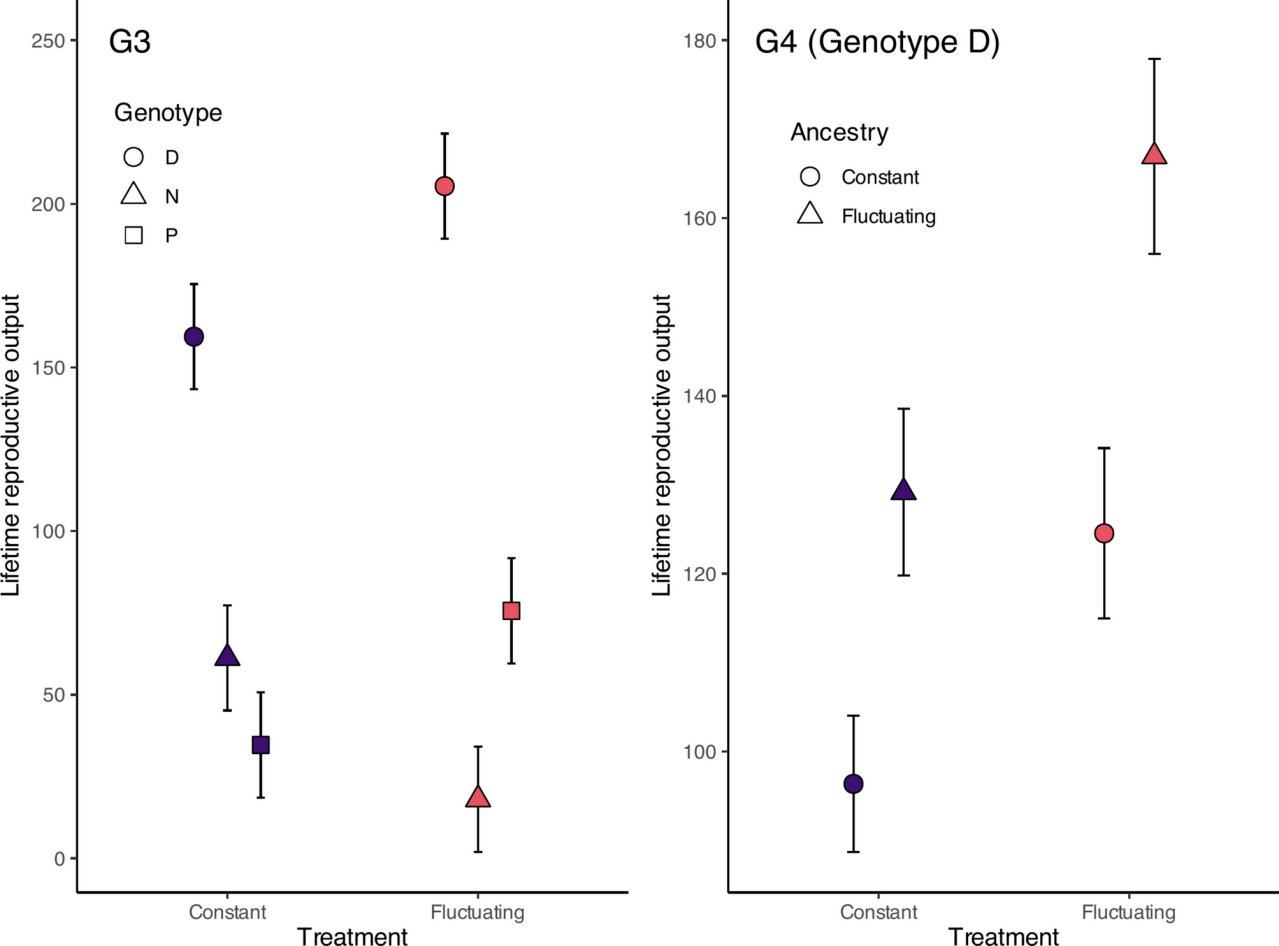
Model estimates (± 1 SE) for reproductive success (total number of neonates per female) for each treatment regime. Generation G3 displays the treatment group and genotype with the shape denoting the genotype and the colour representing the treatment factor. Generation G4 displays the effects of the current and ancestral treatments within genotype D.

The two-way interaction between the treatment group and the genotype significantly affected the number of offspring produced within the G3 generation (Table 1; Fig. 3). More precisely, I found that genotype D was unaffected by the treatment differences, yet was significantly more productive than genotype P when under constant UVR but not under fluctuating UVR. When exposed to fluctuating UVR genotype D had significantly more offspring than genotype N (Table 3).

**Table 3:** Tukey HSD pairwise comparisons of reproductive output between treatment regimes (constant or fluctuating) and genotypes (D, N, or P) of the G3 generation.

Contrast	Ratio	S.E	t.ratio	*p*-value
D: Constant–N: Constant	3.13	1.19	1.86	0.45
D: Constant–P: Constant	**9.13**	**5.59**	**3.61**	**0.016**
D: Constant–D: Fluctuating	0.78	0.48	− 0.41	1
N: Constant–P: Constant	2.92	1.79	1.75	0.52
N: Constant–N: Fluctuating	3.55	2.18	2.07	0.34
P: Constant–P: Fluctuating	0.3	0.18	− 1.97	0.39
D: Fluctuating–N: Fluctuating	**14.23**	**8.72**	**4.33**	**0.003**
D: Fluctuating–P: Fluctuating	3.5	2.14	2.04	0.35
N: Fluctuating–P: Fluctuating	0.25	0.15	− 2.289	0.24

Significant values are in bold.

**Fig. 4:**
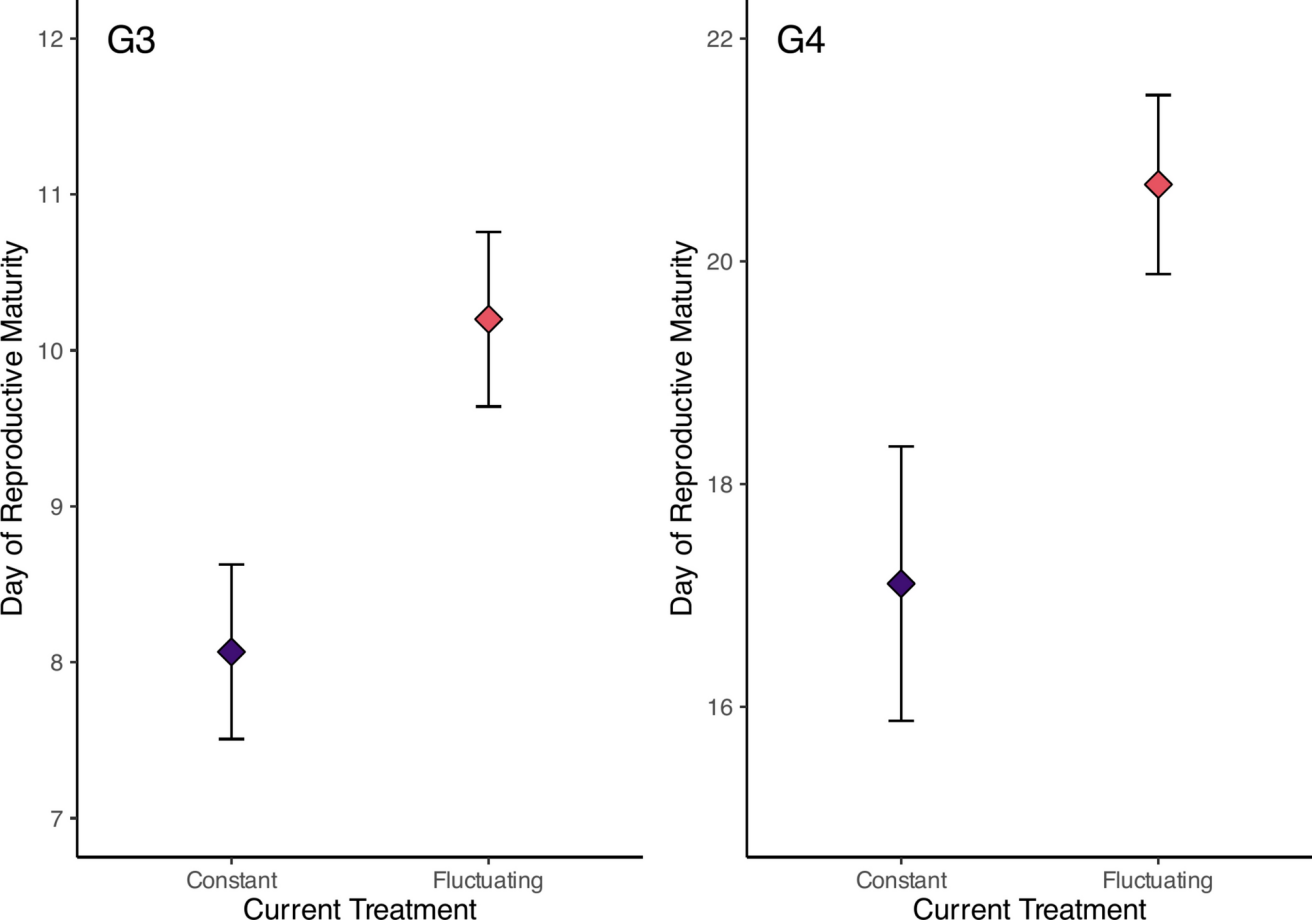
The average day at which individuals become reproductively mature in each treatment regime. The points display the mean ± 1 SE.

Increased variability of the fluctuating UVR regime delayed the date of reproductive maturity in the G3 generation (Table 1; Fig. 4). Genotype also emerged as a significant determinant of reproductive maturity (Table 1; Supplementary Fig. S5). No interaction was found between the treatment and genotype. Averaging across all genotypes, individuals in the constant treatment achieved reproductive maturity at day 8.1 (± 0.56 SE), whereas those in the fluctuating treatment became reproductive at day 10.2 (± 0.56 SE). When averaging over treatment group, genotype D was the fastest to mature at 6.6 (± 0.69 SE) days and the genotype P was the slowest, taking nearly twice as long (12.6 ± 0.69 SE).

### Generation G4

**Fig. 5:**
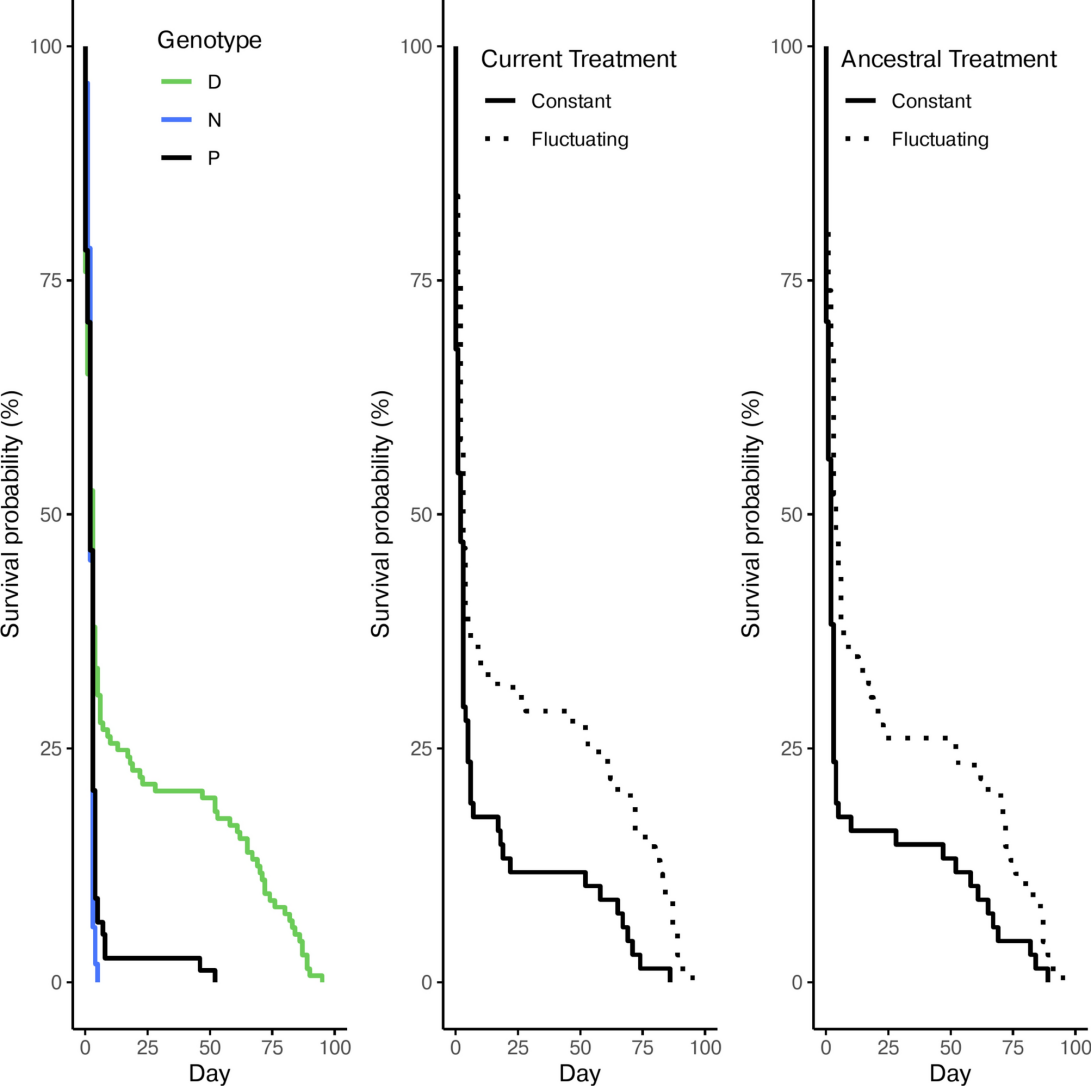
Survival curves of the G4 generation displaying the effects of genotype, current treatment and ancestral treatment in the respective panels from left to right. The centre and rightmost panels refer only to genotype D.

Survival in generation G4 was determined by the current treatment and ancestry independently, i.e., no interactions were detected (Table 1). There was an obvious disparity between genotypes in length of survival, which violated the models’ assumptions, therefore I modelled each genotype separately. Genotype D displayed that individuals under a currently fluctuating environment had a 54.07% higher survival rate irrespective of ancestry (Fig. 5; Table 1). Despite the lack of a significant interaction, ancestral treatment also increased the survival rate. Individuals that had a mother in a fluctuating environment had a 54.81% higher chance of survival (Fig. 5). There were no differences due to either current treatment or ancestry for both genotypes N and P (Fig. 5; Supplementary Table S2).

Treatment and Ancestry significantly interacted to determine the number of offspring produced in generation G4 (Table 1, Fig. 3). It is important to note here, that this only represents one genotype. In large part, this is due to no individuals of genotype N reproducing, and only two individuals of genotype P, both of which were in the fluctuating UVR treatment group. This means that genotype N and P were not significantly different from zero whereas genotype D averaged 67.9 (± 55.3 SE) offspring per individual. Within this genotype, those in the fluctuating UVR treatment group averaged 77.2 (± 62.9 SE) offspring per individual whereas those in a constant UVR environment averaged 59.7 (± 48.6 SE).

**Fig. 6:**
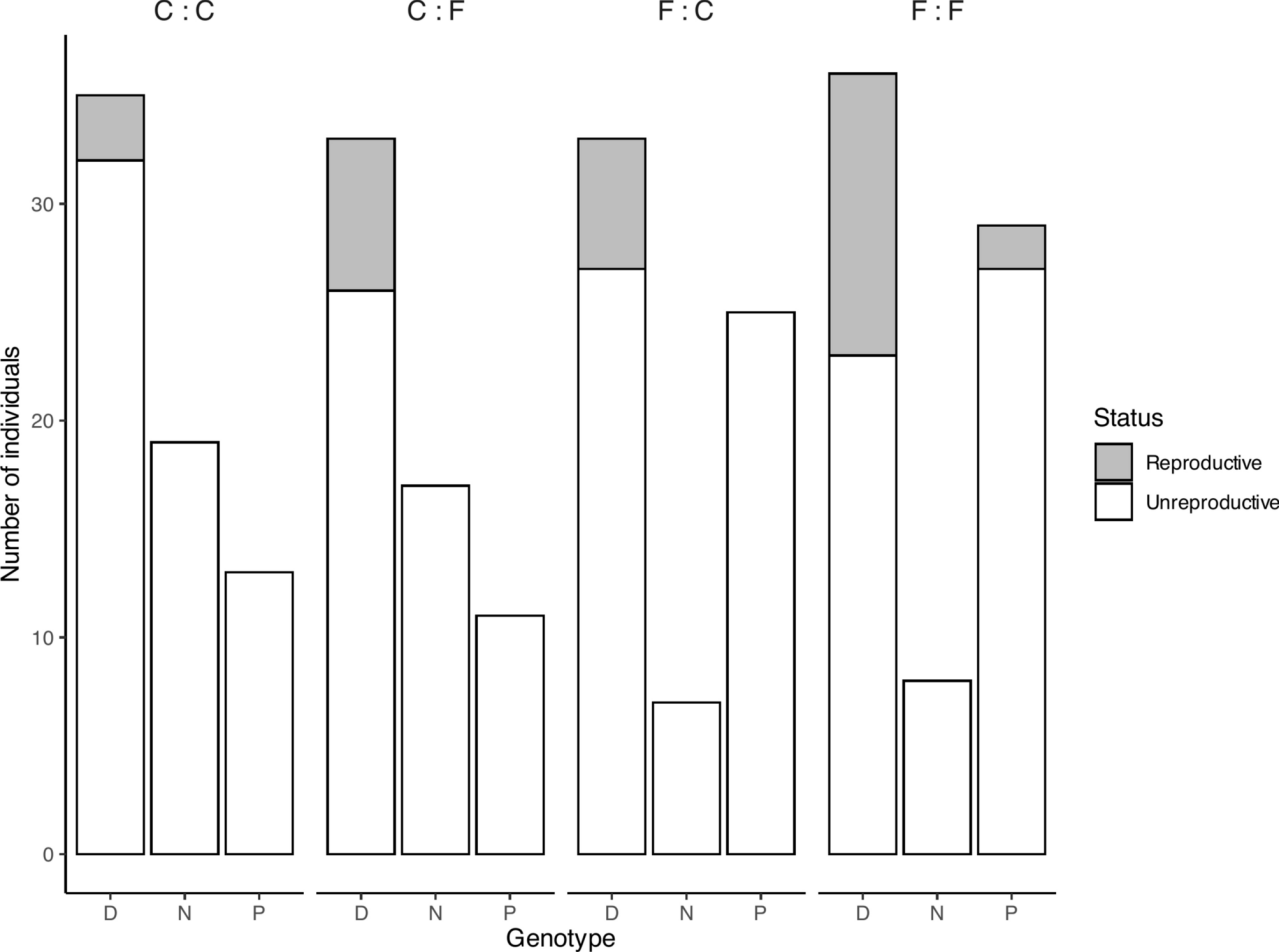
Frequency of generation G4 individuals that became reproductively mature by genotype and ‘Ancestral regime : Current regime’ combinations. ‘C’ represents ‘Constant’ and ‘F’ represents ‘Fluctuating’ regimes.

Current treatment regime significantly delayed the date of reproductive maturity by 3.8 days in the fluctuating regime as compared to the constant regime (Fig. 4). Neither ancestry, nor the interaction between ancestry and the current treatment group had an impact on the date of reproductive maturity (Table 1). As date of maturity was determined only in the one genotype, I also examined the frequency of reproductive and unreproductive individuals across all genotypes. There was a strong association between the number of individuals that became reproductive in the generation G4, ancestry, current treatment and genotype (M^2^ = 21.15, *d.f.* = 6, *p* = 0.002) which is evident in Fig. 6. In the fluctuating treatment 16.4% of individuals became reproductive, in comparison with only 6.8% in the constant treatment. This was similar in magnitude to the ancestral treatment with 15.2% of individuals with a mother in the fluctuating environment becoming reproductive as opposed to 7.8% of those with mothers in the constant treatment group. Genotype, however, shows the largest disparity with 21.2% of genotype D, 2.6% of genotype P and no genotype N becoming reproductive. When binned according to whether the mothers and daughters experienced the same- or different regimes, those experiencing the same environments (irrespective of which) showed higher numbers of reproductive individuals (Supplementary Fig. S6).

## Discussion

All threats, including predation risk, starvation and UVR exposure may affect an individual organism in either a more consistent or fluctuating manner. Whereas the theoretical impact of stressor variation on an organism’s evolutionary strategy is well-established^1,15,22^, our empirical understanding of the consequences of such fluctuations in stressors, independent of the intensity, remains limited. In this study, I show that exposure to different temporally variable UVR regimes have significant fitness consequences in multiple life history traits. I hypothesised that constant exposure would have the greatest fitness advantage, due to the predictable nature of the stressor. Contrary to my initial predictions, based upon prior evidence^43^, these results suggest that fluctuating UVR regimes lead to higher fitness compared to those exposed to an equally intense but more constant UVR regime. Furthermore, these effects carryover to the following generation which does not support the predicted fitness advantages from the maternal matching hypothesis.

Previous studies have found many potential adverse effects of UVR^28,33,50^, and have even shown how the effects span multiple generations^10^. In generation G3, my results indicate that survival and reproduction are strongly influenced by the interaction between UVR variability and the clonal line, whereas maturity was simply affected by the treatment and genotype independently. This implies that certain genotypes could be evolutionarily predisposed to differences in UVR variability, but this remains untested. Despite this, each line used here originated in a geographically similar area but the physical, chemical and biological properties of these lakes are likely to have defined differences. In particular, the UVR extinction depth is a viable proxy for the threat posed by UVR. Of the clones used, two of the three originated from ponds with a measured UV profile (genotype D from lake Bysjön and genotype N from lake Fnifahosjön). Lake Bysjön in comparison to lake Fnifahosjön is more transparent, thereby allowing UVR to penetrate deeper and potentially damage zooplankton, yet *Daphnia* in lake Bysjön were still visible at the water surface during mid-day (personal observations). As would be hypothesised, genotype D was better adapted to UVR stress than genotype N. However, this should be treated as anecdotal as there is no UVR profile for the native pond of genotype P, nor were multiple clones from each lake used. Future studies could directly address this question of UVR adaptation through comparisons of within- and between-lake clonal fitness, along a gradient of increasing transparency. Nonetheless, the various inter-clonal responses not only demonstrate the importance in utilising multiple genotypes in experimental studies, but also highlight genotype-by-environment interactions that may be a key to explain the incredibly rapid local adaptations by many zooplankton, including *Daphnia*^59,60^.

Generation G4 results reveal current treatment is a significant determinant of survival, reproduction and maturity, with those in the fluctuating environments exhibiting longer lifespans, increased offspring production, and delayed reproductive maturity. It is worth mentioning that genotype also impacts survival, but there were no a priori hypotheses as to which genotype would fare better. While ancestry did not exhibit any interactions with current treatment as predicted by the maternal matching hypothesis, it emerged as a significant contributor to survival and reproduction, following the same pattern as the current treatment. In addition to these significant differences in survival, it is evident that generation G4 suffered far greater mortality early in life across both ancestries as compared with the mortality in generation G3. Given generation G4 and G3 were largely contemporaneous, this indicates the presence of negative carryover effects of UVR, as opposed to a difference in the stress, covarying with time. Such carryover effects have been demonstrated in other multigenerational studies^10^. Taken together, this suggests that those mothers from a fluctuating environment were better able to allocate resources to all life history traits, which extends across generations to their offspring.

Trade-offs have been identified between various trait combinations, with one of the most extensively studied being the trade-off between early-life reproduction and survival^61^. As the reproductive value of an organisms is typically higher earlier in life, due to chance of death increasing as the organism ages, earlier maturation is often favoured. This investment into reproduction means less energy is available to repair the cellular damage accrued over life, which leads to earlier death^62,63^. As this relies on the perception of both the environmental conditions and the organism’s reproductive value, under a predictably stressful environment an organism may decide to produce offspring earlier. However, as animals likely accrue information through processes similar to Bayesian updating (the combination of prior information and new data leading to decision making)^64^, exposure to variably stressful environments may provide too little information to reliably initiate earlier reproduction. Therefore, a delayed reproduction may be favoured in order to assimilate more accurate information. In this study, I observed the same phenomena of an early maturation date coupled with shortened lifespan, which led to lower fitness values (fewer individuals produced towards the next generation) in the constant UVR environment. Evidently, this marginally earlier maturity did not compensate for the decrease in lifespan. This suggests that the ‘predictability’ of the constant treatment may be an ‘evolutionary trap’^65^ due to the timing of investment in reproduction coupled with the physiological costs of UVR exposure early in life. However, it is important to note here how despite the constant treatment group being potentially ‘predictable’, to accurately disentangle predictability of a stressor and the period of stress requires a carefully considered experimental design that also includes regular fluctuations. This would allow the disambiguation of predictable stress from the consistency of stress.

Expanding on the physiological costs incurred by UVR, such as the increase in malonaldehyde concentration and catalase activity^66^, even repair mechanisms may negatively impact fitness if produced at high doses^22^. In zooplankton, UVR triggers a range of responses, including an increase in metabolism, engagement of antioxidant pathways, and upregulation of photorepair mechanisms/compounds, such as the photo-enzymatic repair (PER) process that utilizes the photolyase enzyme^34,49^. PER is specifically induced by UV-A radiation, which is the predominant wavelength emission used in this study, and acts by repairing cytotoxic photoproducts^67,68^. Although studies have demonstrated interspecific differences in PER activity and how various developmental stages rely on this mechanism to different degrees, no study has quantified how short-term fluctuations of UVR and photosynthetically active radiation (i.e., visible light) affect this response, and specifically the dose produced. Plainly, further research is needed in relation to the fluctuations and duration of UVR on the physiological repair mechanisms, in order to mechanistically link PER and the fitness outcomes observed. Despite this, and the fact that PER is a relatively slow reaction^49^, it has been stated that even short periods, on the minute-scale, can be enough to increase survival after UVR exposure^33^. Perhaps, the earlier onset of UVR coupled with short (15–60 min) periods of PER activating visible light in the fluctuating treatment in my study were then capable of offsetting negative effects of UVR, compared to the single block of UVR in the constant regime. Furthermore, this appears more critical in early life, in line with findings that report UVR-induced early life mortality^28^ and the differential effectivity of PER at distinct developmental stages^69^.

Such UVR-induced early life mortality, which is clear in generation G4, may explain the disparity in results and original hypothesis based on within generation fitness consequences of fluctuating UVR^43^. More specifically, Stábile et al.^43^ isolated juvenile (8 day) *Daphnia magna* for the study whereas I used day-old neonates. Therefore, the early life selection, which I show here is greater in the constant environment, was missed. This highlights the importance of timing of the novel environmental conditions; if the variable stressor arises in adulthood, it may reduce current reproductive output, yet if that stress continues into the next generation the offspring may do better than if they were exposed to a more consistently stressful environment. Despite differences between the study by Stábile et al.^43^ and this study regarding the within generation response, a common result is that the focal genotypes have distinctly different responses.

In a broader context, my study highlights that genotype-by-environment, i.e. eco-evolutionary, interactions have profound effects on the clonal representation in the environment, and I show here that among only three genotypes, there are highly different responses to the variability or periodicity of the mortal threat UVR. Even within the confines of this four-generation study one genotype became extinct, and another had only two individuals in generation G4 that reproduced (Fig. 6). As seasons change in natural ecosystems, the fluctuations of stressors like UVR may also vary, which could lead to local extinction of genotypes, as demonstrated here. Furthermore, as cloud cover is moving poleward due to climate change^70^ my results indicate that *Daphnia magna* will likely be under stronger selection due to increased UVR, especially considering the predicted increase in heat waves will bring a more consistent period of UVR exposure^71^. Alternatively, the serious fitness consequences of UVR may facilitate the evolution of specialised strategies, leading to patterns of seasonal clonal succession. In support of this notion is the documented clonal composition variation of zooplankton in response to variation in multiple stressors including toxic food and predation cues^60,72,73^.

In conclusion, I demonstrate that the variability of UVR exposure has fitness consequences that are independent of the total dose of irradiation. These results suggest that organisms experiencing fluctuating stressors may deploy adaptive strategies that mitigate fitness losses, in contrast to those exposed to constant stressors, which may amplify fitness consequences. Furthermore, these environments have intergenerational consequences, with prior selective pressures shaping genotypic responses to new challenges. This underscores the role of temporal variability in driving shifts in seasonal clonal successions and determining dominant genotype–phenotype combinations. By considering multiple generations, diverse genotypes, and both stable and variable environmental regimes, this study highlights the nuanced mechanisms through which organisms navigate variable stressors, advancing our understanding of evolutionary strategies in dynamic environments and providing empirical data for the further development of theory on dynamic environments.

## Supplementary Information

Supplementary Information.

## Data Availability

Data and code used in this study are available at the Dryad (10.5061/dryad.m63xsj4cw) and Zenodo (10.5281/zenodo.14500807) repositories, respectively.
