# Supplementary material for: Variable stressor exposure shapes fitness within and across generations
Source: Sci Rep. 2025 Jan 29;15:3626. doi: 10.1038/s41598-025-87334-8 (PMC11779894; doi:10.1038/s41598-025-87334-8)
Supplement: Supplementary file 1 — Supplementary Information. [file 41598_2025_87334_MOESM1_ESM.docx]

Supplementary information for
‘Variable stressor exposure shapes fitness across generations’

Marcus Lee^1,2^

^1^Aquatic Ecology, Department of Biology, Lund University, Lund, Sweden

^2^Department of Biology, University of Texas at Arlington, Arlington, USA

ORCID: 0000-0002-3320-3010

[marcus.lee@biol.lu.se](mailto:marcus.lee@biol.lu.se)

Table S1. Sample sizes used for statistical analyses in the G4 generation

| Ancestry | Treatment | Genotype | Variable | Sample size |
| --- | --- | --- | --- | --- |
| Constant | Constant | D | Lifespan | 35 |
|  |  | N |  | 19 |
|  |  | P |  | 13 |
|  |  | D | Reproductive output | 35 |
|  |  | N |  | NA |
|  |  | P |  | NA |
|  |  | D | Maturity day | 3 |
|  |  | N |  | NA |
|  |  | P |  | NA |
|  |  | D | Reproductive status | 33 |
|  |  | N |  | 7 |
|  |  | P |  | 25 |
| Constant | Fluctuating | D | Lifespan | 33 |
|  |  | N |  | 17 |
|  |  | P |  | 11 |
|  |  | D | Reproductive output | 33 |
|  |  | N |  | NA |
|  |  | P |  | NA |
|  |  | D | Maturity day | 7 |
|  |  | N |  | NA |
|  |  | P |  | NA |
|  |  | D | Reproductive status | 33 |
|  |  | N |  | 17 |
|  |  | P |  | 11 |
| Fluctuating | Constant | D | Lifespan | 33 |
|  |  | N |  | 7 |
|  |  | P |  | 25 |
|  |  | D | Reproductive output | 33 |
|  |  | N |  | NA |
|  |  | P |  | NA |
|  |  | D | Maturity day | 6 |
|  |  | N |  | NA |
|  |  | P |  | NA |
|  |  | D | Reproductive status | 33 |
|  |  | N |  | 7 |
|  |  | P |  | 25 |
| Fluctuating | Fluctuating | D | Lifespan | 36 |
|  |  | N |  | 8 |
|  |  | P |  | 29 |
|  |  | D | Reproductive output | 36 |
|  |  | N |  | NA |
|  |  | P |  | NA |
|  |  | D | Maturity day | 14 |
|  |  | N |  | NA |
|  |  | P |  | NA |
|  |  | D | Reproductive status | 36 |
|  |  | N |  | 8 |
|  |  | P |  | 29 |


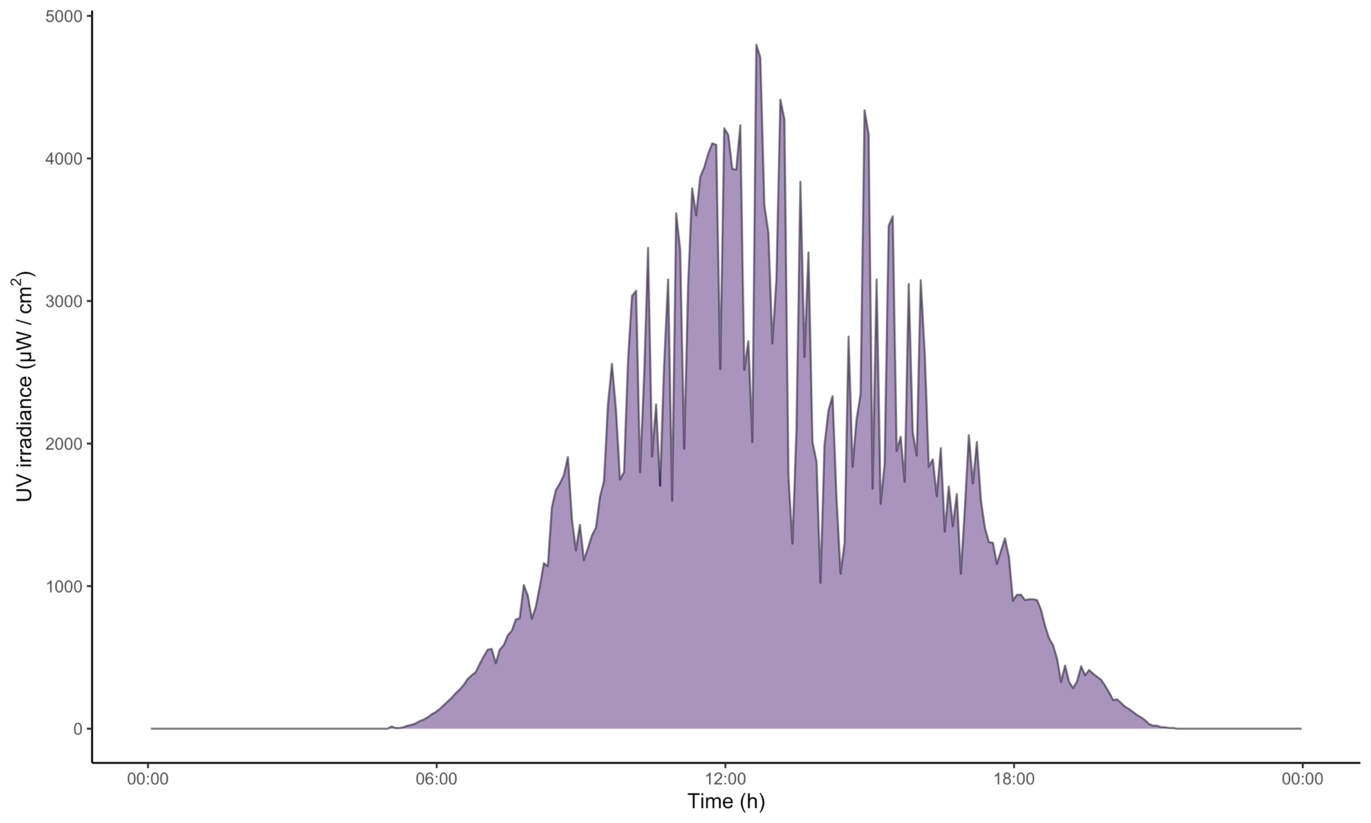


Figure S1. Natural variation in solar UV-A radiation over a random day (August 8^th^ 2021). Data collected from a UVMICROLOG (sglux GMbH, Germany) placed at water surface outside Ekologihuset, Lund University (N 55.713809, E 13.208191).


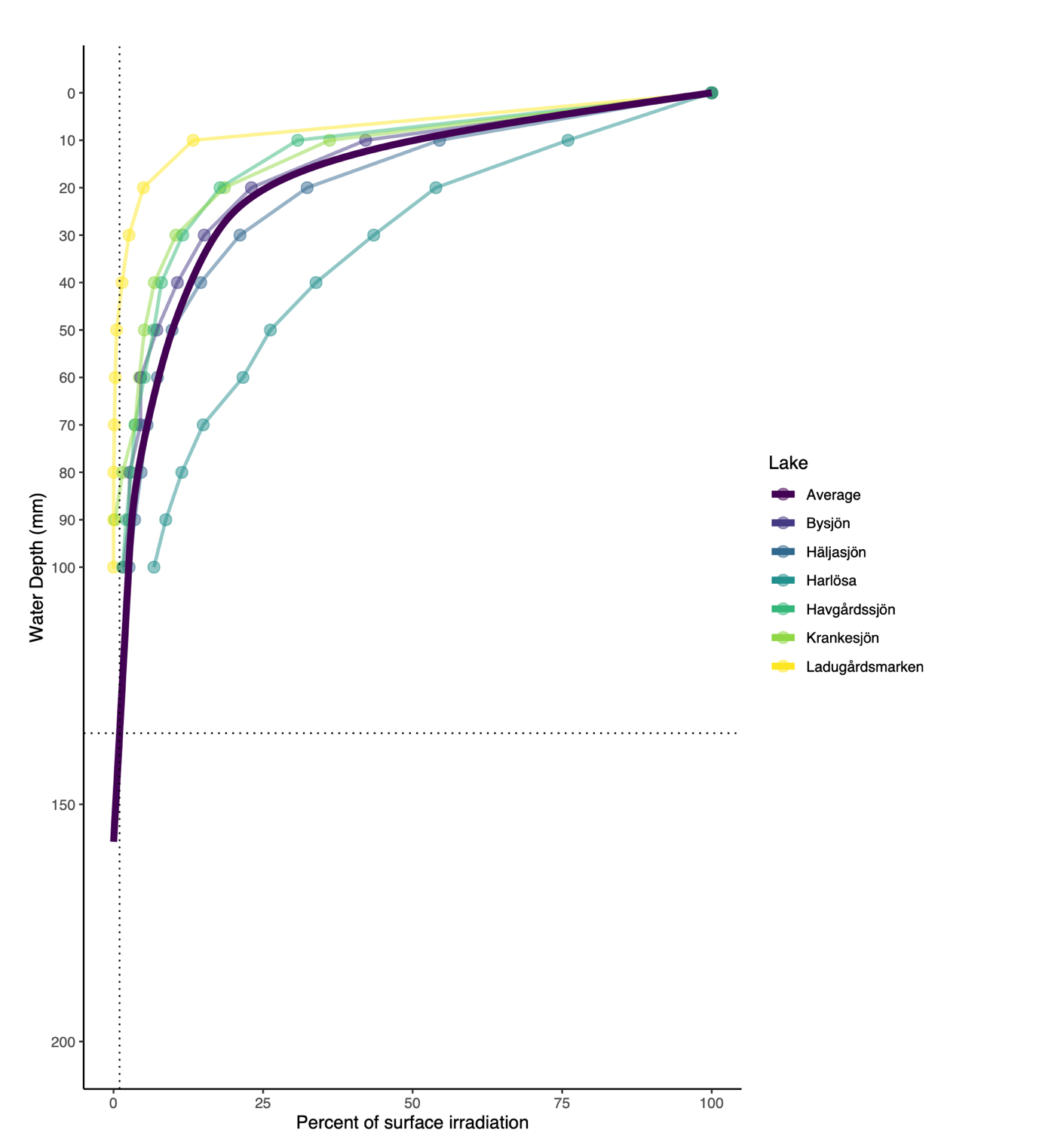


Figure S2. The UV-A extinction rate of 6 lakes in southern Sweden, performed over a 1-meter depth. The level of UVR used in the experiment is represented by the vertical dotted line and the estimated depth at which this UVR level would be expected is denoted by the horizontal dotted line. The predicted average is the dark purple solid line.


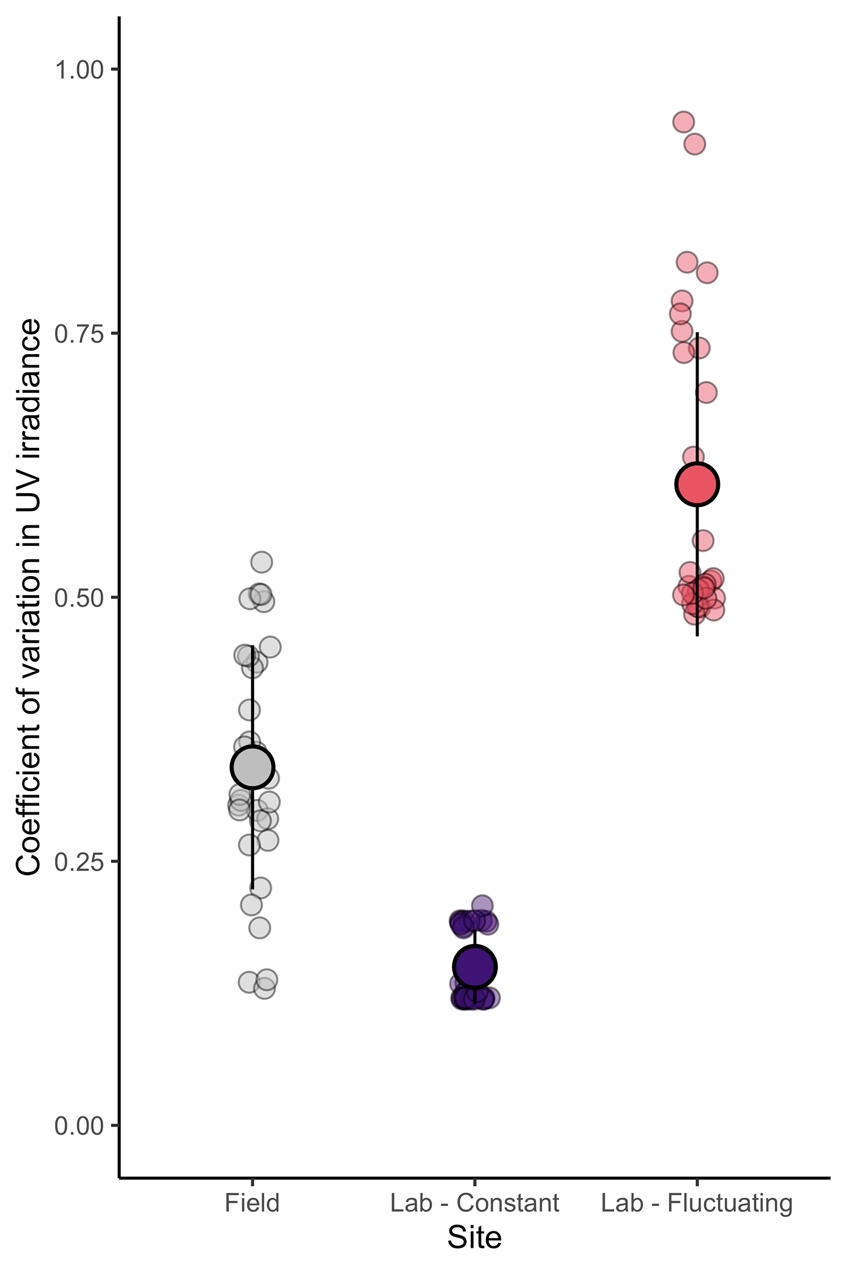


Figure S3. The coefficient of variation of UV-A irradiance, not intensity, in both laboratory treatments and natural solar radiation. Data collected from UVMICROLOG (sglux GMbH, Germany) UV-A data loggers. The data presented relates only to 10:00 – 16:00 each day in August 2021 as to compare the variation in UV-A radiation during the experimental period. This demonstrates the successful design of the experiment with a clear difference between the lab treatments, and also that they mirror the near extremes of natural variation in solar radiation.

Figure S4. A schematic of the lighting schedule for UV lights with yellow representing white light only and purple representing both white light and UV light. A) refers to the constant environment with UVR being present in one long block, whereas B) refers to the fluctuating UVR schedule, whereby UVR potentially changing every 15 minutes. To prevent *Daphnia* becoming accustomed to the same pattern of UVR, the schedule was changed between B1, B2, B3 and B4 randomly every 1-4 days (randomly decided by the role of a four-sided die).

Figure S5. The average day at which individuals in generation G3 become reproductively mature for each genotype. The points display the mean ± 1 SE.

Table S2. Results from the non-significant survival models for G4 genotypes N & P

| Genotype | Explanatory variable | d.f. | χ^2^ | p value |
| --- | --- | --- | --- | --- |
| N | CurrentTreatment | 1 | 0.0453 | 0.8315 |
|  | Ancestry | 1 | 0.5687 | 0.4508 |
| P | CurrentTreatment | 1 | 1.4571 | 0.2274 |
|  | Ancestry | 1 | 2.4819 | 0.1152 |

Figure S6. Frequency of generation G4 individuals that became reproductively mature by genotype and whether the individual was in the same or different UVR regime as their mother. I.e., ‘Constant : Fluctuating’ and ‘Fluctuating : Constant’ constitute ‘Different’ whereas ‘Constant : Constant’ and ‘Fluctuating : Fluctuating’ make up the ‘Same’ category.
